# A Brief Incubation of Cumulus-Enclosed Mouse Eggs in a Calcium-Free Medium Containing a High Concentration of Calcium-Chelator Markedly Improves Preimplantation Development

**DOI:** 10.3390/ijerph17103505

**Published:** 2020-05-17

**Authors:** Valeria Merico, Silvia Garagna, Maurizio Zuccotti

**Affiliations:** Department of Biology and Biotechnology Lazzaro Spallanzani, University of Pavia, 27100 Pavia, Italy; valeria.merico@unipv.it (V.M.); silvia.garagna@unipv.it (S.G.)

**Keywords:** In vitro fertilization, cumulus cells, oocyte, preimplantation, mouse

## Abstract

The presence of cumulus cells (CCs) surrounding ovulated eggs is beneficial to in vitro fertilization and preimplantation development outcomes in several mammalian species. In the mouse, this contribution has a negligible effect on the fertilization rate; however, it is not yet clear whether it has positive effects on preimplantation development. Here, we compared the rates of in vitro fertilization and preimplantation development of ovulated B6C3F1 CC-enclosed vs. CC-free eggs, the latter obtained either after a 5 min treatment in M2 medium containing hyaluronidase or after 5–25 min in M2 medium supplemented with 34.2 mM EDTA (M2-EDTA). We found that, although the maintenance of CCs around ovulated eggs does not increment their developmental rate to blastocyst, the quality of the latter is significantly enhanced. Most importantly, for the first time, we describe a further quantitative and qualitative improvement, on preimplantation development, when CC-enclosed eggs are isolated from the oviducts in M2-EDTA and left in this medium for a total of 5 min prior to sperm insemination. Altogether, our results establish an important advancement in mouse IVF procedures that would be now interesting to test on other mammalian species.

## 1. Introduction

In most eutherian mammals, the metaphase II oocyte (egg) is ovulated, surrounded by layers of cumulus cells (CCs) embedded in an extracellular matrix of hyaluronic acid. CCs play several crucial roles. They are the first barrier that sperm encounter when gaining the ampulla region in their travel towards the egg, physically entrapping the male gametes and guiding them to the egg [1,2,3]. Two studies proved that mouse sperm initiate the acrosome reaction during their passage through the CCs [4,5,6], rather than later when entering in contact with the zona pellucida, as widely thought before [7,8,9]. Additionally, a number of studies have discussed a function for CCs in the selection of morphologically normal human sperm [10,11,12]. Although the presence of CCs surrounding the egg is not strictly required for successful in vitro fertilization (IVF), suggesting that they are dispensable, a number of studies have underlined the beneficial effect of their presence on both rates of fertilization and preimplantation development in pigs [13,14,15], buffalos [16], and cattle [17,18,19,20,21,22]. In the mouse, the presence of CCs surrounding ovulated eggs has a negligible effect on the in vitro fertilization rate [23], but it still remains unclear whether they are required at the time of fertilization for improved preimplantation developmental competence or if the procedure with the enzyme hyaluronidase, routinely used for the removal of CCs, may result in a drawback of their early developmental potential.

In the present study, we set out 1) to compare fertilization and preimplantation development rates of ovulated B6C3F1 CC-enclosed eggs with those of CC-free eggs, the latter obtained after a treatment either in M2 medium containing hyaluronidase or, to avoid the use of this enzyme, in Ca-free M2 medium supplemented with 34.2 mM of the calcium-chelating agent EDTA (ethylene-diamine-tetraacetic acid), a concentration used for the disaggregation of mouse preimplantation embryo blastomeres [24]. CCs were removed completely after 25 min EDTA treatment; however, already after 5 min we could observe—under the microscope at 200× magnification—a relaxation without cell loss of the cumulus oophorus. Thus, 2) we tested the hypothesis that a relaxation of the CC-to-CC contacts, obtained thanks to a brief treatment in M2 medium containing a high metal ion-chelator concentration, could result in an improvement of the developmental outcome.

At completion of these experiments, we discovered that although the presence of CCs around ovulated eggs did not improve their developmental rate to blastocyst, the quality of the latter was significantly enhanced. Importantly, we demonstrated that a 5 min EDTA treatment of CC-enclosed eggs prior to insemination, whilst preserving cumulus oocyte complexes (COCs) integrity, remarkably enhanced, compared to all the other treatments tested, their developmental competence to blastocyst.

## 2. Materials and Methods

### 2.1. Animals and Reagents

Four 5-week-old female and 6-month-old male B6C3F1 mice were purchased from Charles River (Como, Italy). Animals were maintained under controlled room conditions (22 °C, with 60% air moisture, and 12L:12D photoperiod). The experiments of this study conducted on animals were performed in accordance with the guiding principles of European (n. 86/609/CEE) and Italian (n. 116/92, 8/94) laws protecting animals used for scientific research and were approved by the Italian Health Ministry with the project identification code N. 1100/2016-PR (15 November 2016). All chemicals used were purchased from Sigma-Aldrich (St. Louis, MO, USA), unless otherwise stated. Ultrapure MilliQ water (Merck) was used for preparing media.

### 2.2. Oocytes Isolation, In Vitro Fertilization and Preimplantation Development

Females were injected with 3.5 I.U. Folligon (Intervet Srl, Italy) followed, 48 h later, by an injection of 3.5 I.U. Corulon (Intervet), and the oviducts were isolated after 15 h to collect ovulated metaphase II (MII) eggs. Following isolation, ovulated CC-enclosed eggs were released from the oviducts in 1 mL of M2 medium in its regular formulation not containing EDTA [25], then they were immediately transferred in M2 medium with the hereafter reported changes in formulation, corresponding to the different tested experimental groups, to obtain either CC-enclosed (i–iv) or CC-free (v–vii) eggs: i) M2 medium (control, CTRL); ii) Ca^2+^-free M2 medium for 5 min (M2-Ca^2+^-free); iii) Ca^2+^-free M2 medium supplemented with 34.2 mM (10 mg/mL) EDTA for 5 min (M2-EDTA^5^); iv) Ca^2+^-free M2 medium supplemented with 26.3 mM (10mg/mL) EGTA for 5 min (M2-EGTA); v) M2 medium containing 500 I.U. hyaluronidase type II for 5 min (M2-Hyal); and vi) Ca^2+^-free M2 medium supplemented with 34.2 mM (10 mg/mL) EDTA for 25 min (M2-EDTA^25^).

Then, CC-enclosed or CC-free oocytes were washed twice in 1 mL M2 medium and twice in Whittingham medium [25] where they were inseminated with capacitated sperm.

Sperm were isolated as previously described [26] and incubated for 60 min in 100 μL drops of Whittingham medium, supplemented with 0.1 mM EDTA, at a final concentration of 1.8 × 10^6^ sperm/mL. Two hours after insemination, groups of CC-free or CC-enclosed oocytes were transferred into a fresh Whittingham drop (2 µL/oocyte) for another hour and then the presumptive zygotes were transferred into M16 medium supplemented with 0.1 mM EDTA (2 µL/oocyte) for preimplantation development. To define the fertilization rate, zygotes were observed 6 h after insemination under an inverted microscope; those with two pronuclei and at least two polar bodies were further cultured in M16 medium. Development to the 2-cell stage was evaluated at 24 h post-insemination (p.i), to morula at 72 h p.i. and to the blastocyst stage at 96 h p.i.

### 2.3. Immunocytochemistry

Blastocysts, obtained from five independent experiments each with its own CTRL group, were fixed with freshly prepared 4% paraformaldehyde for 30 min and then permeabilized with 0.5% Triton X-100 for 15 min. To suppress nonspecific antibodies binding, embryos were incubated with 0.5% blocking reagent (Roche) in TNT buffer (0.1 M Tris-HCl, pH 7.5, 0.15 M NaCl, and 0.05% Tween-20) for 20 min at 4 °C. Embryos were processed for consecutive immunolabeling using a rabbit polyclonal antihuman OCT4 (Abcam, cat. no. ab19857, diluted 1:400 in PBS) and a rabbit antihuman CDX2 (Cell Signaling Technology, cat. no. 3977S, diluted 1:100 in PBS) antibodies. Because the primary antibodies were raised in the same species, sequential detection of both epitopes was done. Briefly, blastocysts were first incubated for the detection of the OCT4 protein (using the secondary Alexa Fluor488 anti-rabbit IgG, diluted 1:500 in PBS plus 0.01% Tween20, Molecular Probes) and then incubated for the detection of the CDX2 protein (using the secondary Alexa Fluor555 anti-rabbit IgG, diluted 1:500 in PBS plus 0.01% Tween20, Molecular Probes). Both primary and secondary antibodies were applied for 1 h at 37 °C; after each incubation step, embryos were washed through three changes of TNT for 15 min each at 4 °C. Then, embryos were stained with DAPI (0.2 µg/mL in PBS, 5 min), mounted in Vectashield (H-1000, Vector), and slightly pressed to facilitate the determination of the cell number. Three-dimensional images were acquired with an Olympus Provis epifluorescence microscope equipped with single-bandpass filters for DAPI, AlexaFluor 488, AlexaFluor 555, and a Tango motorized Z-axis (MärzhäuserWetzlar, Germany), and a Photometrics CH-350 camera. A collection of optical sections (0.5 μm Z-spacing) was analyzed for cell counting using the Olympus Cell sens Dimension software. We did not use a threshold level of signal but, in order to reduce the variability, the compared blastocysts were processed in the same immunocytochemistry experiment and the images were acquired at the same exposure conditions. Images were merged using Image*J* (http://imagej.nih.gov/ij/) and Adobe Photoshop CS3 software (Adobe, San Jose, CA, USA).

### 2.4. Statistical Analysis

Data, obtained from at least six independent experiments, were analyzed by Student’s *t*-test when the comparison was done between two conditions or by one-way ANOVA when the comparison was performed among more than two conditions. In the presence of a significant difference after the one-way ANOVA a post-hoc test (Fisher LSD Method) was performed. Differences were considered significant when *p* < 0.05.

## 3. Results

In a first set of experiments, our aim was to figure out whether the presence of CCs around the egg was beneficial to fertilization and developmental competence. Thus, we compared the fertilization and development rates of CTRL vs. CC-free eggs, the latter obtained either after hyaluronidase treatment or 25 min culture in M2-EDTA^25^. Following insemination, CTRL, M2-Hyal, and M2-EDTA^25^ eggs showed a similar fertilization rate (1-cell embryos; *p* ≥ 0.14) (Table 1). Then, after the first segmentation division, of the three groups, a significantly higher frequency of CTRL embryos reached the 2-cell stage compared to M2-Hyal (*p* = 0.043) and M2-EDTA^25^ (*p* = 0.001) embryos.

From the 2-cell stage onward, CTRL and CC-free eggs attained morula and blastocyst stages at frequencies not significantly different (*p* ≥ 0.214) (Table 1).

The results summarized in Table 1 show that an amount of eggs, comprised between 11.6–18.1%, remained unfertilized; and also, during the first embryonic division, embryos were lost with a frequency that varied depending on the experimental condition tested, in the range of 5.6 ± 6.5% (CTRL), 19.0 ± 7.2% (M2-Hyal) (*p* = 0.002), or 26.7 ± 8.8% (M2-EDTA^25^) (*p* ≤ 0.001), indicating this step as the most critical in development.

In the next set of experiments, we compared the developmental rates of CTRL vs. 5 min EDTA-treated (M2-EDTA^5^) of CC-enclosed eggs.

When compared to CTRL, M2-EDTA^5^ eggs showed a remarkable improvement of both fertilization (*p* = 0.046) and developmental rates (Table 1). Notably, the frequency of M2-EDTA^5^ embryos that progressed to the 2-cell stage and to blastocyst was significantly higher compared to that obtained with CTRL embryos (*p* = 0.037 and *p* = 0.009, respectively).

Then, we tested whether this fertilization and developmental improvement was attributable to the 5 min incubation in the presence of EDTA or to the Ca^2+^-free M2 medium used. When comparing the effects of either the presence (CTRL) or absence of calcium in the M2 medium, we did not record significant differences either for fertilization (*p* = 0.994) or for their developmental rate to blastocyst (*p* = 0.854) (Table 1).

Altogether, these results indicate that it was not the absence of calcium in the isolation M2 medium, but rather the EDTA ions-chelating activity that determined the observed fertilization and development improvements. To test the hypothesis that EDTA was having this positive effect through a Ca^2+^ chelating activity, in a further set of experiments we used EGTA (ethylene-glycol-tetraacetic acid) that has a specific and higher affinity for calcium ions. CC-enclosed eggs were treated for 5 min in Ca^2+^-free M2 medium containing 26.3 mM EGTA (M2-EGTA). As shown in Table 1, the frequencies of eggs that were fertilized and that developed to blastocyst resembled those obtained with eggs incubated in M2-EDTA^5^ (*p* = 0.767 and *p* = 0.678, respectively).

The total number of blastomeres making up a blastocyst, as well as the number of cells constituting its trophectoderm (TE) and inner cell mass (ICM) are features representative of blastocyst quality [27,28]. Ninety-six hours after insemination, embryos were processed for the immunocytochemical localization of CDX2 and OCT4 proteins, markers of TE and ICM cells, respectively. When, in a first set of experiments, the quality of blastocysts obtained from CTRL was compared to that of those obtained from M2-Hyal eggs, the results (Table 2) brought up a significantly (*p* = 0.037) higher total number of cells in the former (53.1 ± 4.4) compared to the latter (50.1 ± 3.3).

This difference is explained with a higher number of ICM blastomeres (13.1 ± 2.0 vs. 11.0 ± 1.7; *p* = 0.009). Instead, CTRL blastocysts appeared with a lower number of total (*p* = 0.001), TE (*p* = 0.004), and ICM (*p* = 0.010) blastomeres, compared to those obtained from M2-EDTA^5^ eggs (Table 2).

## 4. Discussion

The results of our investigation indicate that the maintenance of CCs around the ovulated egg is beneficial to the outcome of mouse in vitro preimplantation development. More specifically, in the comparison between CTRL CC-enclosed vs. CC-free eggs, we observed that the presence of CCs surrounding ovulated eggs whilst negligible on the fertilization rate—confirming an earlier study [11]—it does slightly, but significantly, improve blastocyst quality.

A further marked enhancement was obtained when CC-enclosed eggs were isolated during a 5 min period in M2 medium containing a calcium-chelating agent, either EDTA or EGTA. EDTA is part of many media used for preimplantation embryo culture, including the M16 medium used in our study (0.1 mM). Whilst the regular M2 medium formulation (like the one we prepared) does not include EDTA, a novelty of our study is that we showed that a 5 min passage of ovulated COCs in M2 medium containing a high EDTA concentration, significantly improves the rate of preimplantation development to blastocyst. Thus, following this treatment, we demonstrated a highly significant increase of 2-cell embryos that reached the blastocyst stage, with three out of six experiments in which we attained a 100% development. Furthermore, the blastocysts obtained displayed a higher number of TE and ICM blastomeres, both contributing to the higher total number of blastocyst cells.

Although our study does not identify a mechanism for the recorded developmental improvement, we speculate that the 5 min treatment, though incapable of removing the CCs surrounding the egg, determines their relaxation and may create a favorable environment for sperm selection. In addition, being EDTA an inhibitor of the 3-phosphoglycerate kinase [29], a key glycolysis enzyme active in CCs and involved in oocyte’s aging after ovulation [30], EDTA-treatment of post-ovulatory COCs might delay oocyte aging and contribute to an overall improvement of their developmental competence.

## 5. Conclusions

Altogether, our results highlight how a 5 min incubation step of ovulated CC-enclosed eggs in M2 medium containing a high concentration of a calcium chelator markedly improves preimplantation development both quantitatively and qualitatively. This new method establishes an important advancement in mouse IVF procedures that would be now interesting to test on other mammalian species. If the improvement recorded with the mouse would be confirmed with other model animals such as the cow, its use could be envisaged in human ART (assisted reproductive technologies), where the prevalence of clinical cycles is now performed using the intra cytoplasm sperm injection technique.

## Figures and Tables

**Table 1:** Number (mean % ± s.d.) * of cumulus cells (CC)-enclosed and CC-free eggs that, after fertilization, developed to blastocyst or of embryos that blocked development during the passage from a stage to the next.

	Treatment		MII	Preimplantation Development							
Eggs	Type	Time (min)		Unfertilized	1-Cell	Arrested	2-Cell	Arrested	Morula **	Arrested	Blastocyst **
**CC-enclosed**	CTRL	5	147	21	126	6	120	10	110	7	103
				(12.7 ± 6.3) ^a,b^	(87.3 ± 6.3) ^a,b^	(5.6 ± 6.5) ^a^	(82.4 ± 7.5) ^a,b^	(8.8 ± 5.8) ^a,b^	(91.2 ± 5.8) ^a,b^	(6.2 ± 4.0) ^a^	(85.4 ± 3.6) ^a^
	M2-Ca^2+^-free		111	16	96	16	79	6	73	6	67
				(12.7 ± 7.4) ^a,b^	(87.3 ± 7.4) ^a,b^	(15.5 ± 8.8) ^b,c^	(74.2 ± 13.6) ^a,c^	(7.6 ± 2.7) ^a,b^	(92.4 ± 2.7) ^a,b^	(8.5 ± 6.1) ^a^	(84.6 ± 7.5) ^a^
	M2-EDTA^5^		122	6	116	3	113	2	111	2	109
				(4.1 ± 3.3) ^b,c^	(95.9 ± 3.3) ^c^	(2.7 ± 3.3) ^a^	(93.3 ± 4.1) ^d^	(1.6 ± 2.7) ^b^	(98.4 ± 2.7) ^b^	(1.5 ± 2.6) ^a^	(96.9 ± 3.4) ^b^
	M2-EGTA		105	3	102	7	95	2	93	2	91
				(2.9 ± 6.4) ^c^	(97.1 ± 6.4) ^c^	(7.1 ± 2.9) ^a,b^	(90.2 ± 5.9) ^b,d^	(2.2 ± 3.1) ^b^	(97.8 ± 3.1) ^b^	(2.7 ± 3.7) ^a^	(95.2 ± 5.2) ^b^
**CC-free**	M2-Hyal	5	144	16	128	25	103	11	92	6	86
				(11.6 ± 9.7) ^a,b,c^	(88.4 ± 9.7) ^a,b,c^	(19.0 ± 7.2) ^c,d^	(71.1 ± 4.1) ^c^	(11.0 ± 7.9) ^a^	(89.0 ± 7.9) ^a^	(6.6 ± 4.2) ^a^	(83.2 ± 3.6) ^a^
	M2-EDTA^25^	25	130	23	107	26	81	7	74	6	69
				(18.1 ± 7.7) ^a^	(81.9 ± 7.7) ^a^	(26.7 ± 8.8) ^d^	(60.4 ± 12.1) ^e^	(13.5 ± 8.7) ^a^	(86.5 ± 8.7) ^a^	(5.9 ± 5.5) ^a^	(81.3 ± 8.0) ^a^

(*): In the same column, different superscript letters indicate a significant difference; (**): The developmental rate was calculated based on the number of 2-cell embryos set at 100%.

**Table 2:** Mean number ± s.d. of blastomeres counted in blastocysts at 96 h p.i. (DAPI) and of cells positive to trophectoderm (CDX2) or inner cell mass (OCT4) immunocytochemical markers. In brackets, the number of blastocysts analyzed for each experimental group is given. The images show a typical blastocyst that developed from a CC-enclosed egg incubated for 5 min in M2-EDTA (M2-EDTA^5^) prior to sperm insemination. In the same column, different superscript letters indicate a significant difference.

Eggs	Treatment (Number of Embryos Analyzed)	DAPI 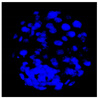	CDX2 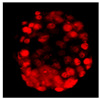	OCT4 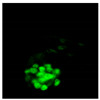	Merge 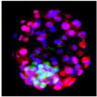
**CC-enclosed**	**CTRL (12)**	53.1 ± 4.4 ^a^	40.2 ± 4.7 ^a^	13.1 ± 2.0 ^a^	
**CC-free**	**M2-Hyal (18)**	50.1 ± 3.3 ^b^	39.1 ± 3.2 ^a^	11.0 ± 1.7 ^b^	
**CC-enclosed**	**CTRL (24)**	52.2 ± 4.3 ^a^	38.7 ± 3.6 ^a^	14.0 ± 1.7 ^a^	
	**M2-EDTA^5^**	65.0 ± 10.8 ^b^	47.1 ± 8.2 ^b^	17.9 ± 4.3 ^b^	
